# Growth and Break-Up of Methanogenic Granules Suggests Mechanisms for Biofilm and Community Development

**DOI:** 10.3389/fmicb.2020.01126

**Published:** 2020-06-03

**Authors:** Anna Christine Trego, Evan Galvin, Conor Sweeney, Sinéad Dunning, Cillian Murphy, Simon Mills, Corine Nzeteu, Christopher Quince, Stephanie Connelly, Umer Zeeshan Ijaz, Gavin Collins

**Affiliations:** ^1^Microbial Communities Laboratory, School of Natural Sciences, National University of Ireland Galway, Galway, Ireland; ^2^Microbial Ecology Laboratory, School of Natural Sciences, National University of Ireland Galway, Galway, Ireland; ^3^Warwick Medical School, University of Warwick, Warwick, United Kingdom; ^4^Infrastructure and Environment, School of Engineering, University of Glasgow, Glasgow, United Kingdom; ^5^Ryan Institute, National University of Ireland Galway, Galway, Ireland

**Keywords:** anaerobic digestion, biofilms, methanogens, microbial communities, sludge granules, wastewater

## Abstract

Methanogenic sludge granules are densely packed, small, spherical biofilms found in anaerobic digesters used to treat industrial wastewaters, where they underpin efficient organic waste conversion and biogas production. Each granule theoretically houses representative microorganisms from all of the trophic groups implicated in the successive and interdependent reactions of the anaerobic digestion (AD) process. Information on exactly how methanogenic granules develop, and their eventual fate will be important for precision management of environmental biotechnologies. Granules from a full-scale bioreactor were size-separated into small (0.6–1 mm), medium (1–1.4 mm), and large (1.4–1.8 mm) size fractions. Twelve laboratory-scale bioreactors were operated using either small, medium, or large granules, or unfractionated sludge. After >50 days of operation, the granule size distribution in each of the small, medium, and large bioreactor sets had diversified beyond—to both bigger and smaller than—the size fraction used for inoculation. Interestingly, extra-small (XS; <0.6 mm) granules were observed, and retained in all of the bioreactors, suggesting the continuous nature of granulation, and/or the breakage of larger granules into XS bits. Moreover, evidence suggested that even granules with small diameters could break. “New” granules from each emerging size were analyzed by studying community structure based on high-throughput 16S rRNA gene sequencing. *Methanobacterium*, *Aminobacterium*, *Propionibacteriaceae*, and *Desulfovibrio* represented the majority of the community in new granules. H2-using, and not acetoclastic, methanogens appeared more important, and were associated with abundant syntrophic bacteria. Multivariate integration (MINT) analyses identified distinct discriminant taxa responsible for shaping the microbial communities in different-sized granules.

## Introduction

Biofilms form in a wide range of natural and built environments (Sutherland, 2001), and have important significance for biogeochemical cycling in Nature (Battin et al., 2016; Flemming and Wuertz, 2019), and further clinical (Arciola et al., 2018) and industrial implications (Jensen et al., 2016). However, though biofilms are classically found as layers, or films, attached to suitable surfaces—from rocks, to medical devices, to ship hulls—aggregation may also occur due to self-immobilization of cells into discrete structures, such as flocs or granules, without the involvement of a surface (Lettinga et al., 1980; Togashi et al., 2014; Wilbanks et al., 2014). Many such examples can be found in engineered environments, such as in biological wastewater treatment, where prevailing conditions of shear, and hydrodynamic, stresses promote flocculation and granulation. Common types include anaerobic ammonium oxidizing (annamox) granules (Kartal et al., 2010), aerobic granules (Beun et al., 1999), and anaerobic (methanogenic) granules (Lettinga et al., 1980), which underpin the success of several high-rate anaerobic wastewater treatment technologies.

Anaerobic granules are small, with diameters generally ranging 0.1–5.0 mm (Ahn, 2000; Batstone and Keller, 2001; Shin et al., 2019), densely-packed biofilm spheres. They comprise a complex microbial community capable of the complete mineralization of organic pollutants through the anaerobic digestion (AD) pathway (Liu et al., 2002; Batstone et al., 2019). The settleabilty of anaerobic granules accounts for long biomass retention—even in “upflow” bioreactors, such as the upflow anaerobic sludge bed (UASB) and expanded granular sludge bed (EGSB) bioreactors, operated with short hydraulic retention times (HRT), and very high volumetric loading and upflow velocities (van Lier et al., 2015). The size distribution of anaerobic granules varies according to wastewater type (Batstone and Keller, 2001), but has also been linked to the hydrodynamics of the digester system (Arcand et al., 1994). Moreover, granule size has been linked to porosity (Wu et al., 2016) and permeability (Afridi et al., 2017)—having further implications for biofilm structure, mass transfer, gas diffusion, and activity (Bhunia and Ghangrekar, 2007; Jiang et al., 2016; Wu et al., 2016; Afridi et al., 2017).

A single granule contains a diverse and dynamic microbial community, capable of adapting to various changes in environmental conditions (McKeown et al., 2009; Cerrillo et al., 2016; Kuroda et al., 2016; Na et al., 2016; Zhu et al., 2017; Keating et al., 2018). Microbial cells are juxtaposed, and immobilized, within a complex matrix of extracellular polymeric substances (EPS) (MacLeod et al., 1995). Within these highly organized consortia, a collection of microbial trophic groups mediates a cascade of interdependent reactions resulting in complete degradation of complex organic wastewater pollutants (Zehnder and Brock, 1980; Dolfing, 1992; Batstone et al., 2019). Equally, the consortium’s species rely on efficient mass transfer of substrates and complex metabolic interdependencies (Embree et al., 2015).

Granulation is a process whereby suspended particles and planktonic cells accumulate, forming small dense biofilm aggregates (Liu et al., 2002; Show et al., 2020). Unlike conventional biofilm formation, which is a well-documented phenomenon (O’Toole et al., 2000; Hall-Stoodley et al., 2004), the specific mechanisms involved in anaerobic sludge granulation are still being teased-out (Sanjeevi et al., 2013; Kim et al., 2014; Gagliano et al., 2017, 2020; Sudmalis et al., 2018; Faria et al., 2019). The topic has been comprehensively reviewed, and the various theories summarized, which can be categorized as physical, microbial, or thermodynamic (Hulshoff Pol et al., 2004; Show et al., 2020). However, none has been solely accepted as a “unified theory on anaerobic granulation” (Liu et al., 2003). The primary consensus seems to be that the genus *Methanothrix* (*Methanosaeta*), a group of acetoclastic methanogens, specifically *Methanothrix soehngenii* (Tindall, 2014), are key organisms during the process (Hulshoff Pol et al., 2004; Show et al., 2020). These archaea can either (i) aggregate together, (ii) attach to suspended particles, or (iii) potentially form a bridge between existing microflocs—aiding in the critical first step of forming granule precursors (Dubourgier et al., 1987; Weigant, 1987; Jian and Shi-yi, 1993). In fact, recent studies still confirm the importance of *Methanosaeta*, even at high salinity (Gagliano et al., 2020).

Many studies have focused on granulation (Show et al., 2020), and associated dynamics of physico-chemical properties and microbial community structure. Fewer studies, however, have addressed the ultimate fate of granular biofilms. Moreover, several studies have reported the spontaneous disintegration of granular biofilms, ultimately leading to process failure (McHugh et al., 2006; Baloch et al., 2008; Kobayashi et al., 2015). Studies into development and fate of granules could help prevent or mitigate such phenomena. Therefore, the primary purpose of this study was to monitor granular growth; to determine whether granules grow and develop in a predictable way, from small to medium and, finally, to large. Díaz et al. (2006) proposed that small granules could be considered “young” and larger granules “old,” or more mature. However, the eventual fate of large, old granules remains unclear. This is the first instance, of which we are aware, when granules have been compartmentalized into size-resolved fractions (small, medium, and large), which were then used to separately start up bioreactors to investigate granule development and fate in such a way. Moreover, undisturbed sludge, providing a “meta-community” and full complement of size fractions, was used as a comparator. The extent, nature and ecology of “new” granules emerging in the experiments was monitored.

## Materials and Methods

### Source and Fractionation of Biomass

Anaerobic sludge was obtained from a full-scale (8256 m^3^), mesophilic (37°C), EGSB bioreactor in the Netherlands treating potato-processing wastewater. The full-scale bioreactor was operated at an upflow velocity of 1.2 m h^–1^ and an HRT of 6.86 h.

**FIGURE 1:**
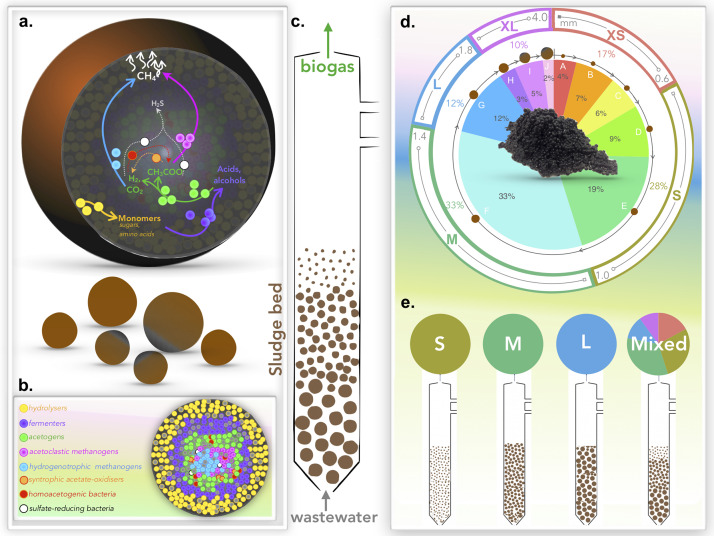
Schematics illustrating: **(a)** the AD pathway of organic matter degradation in the context of a granule; **(b)** theoretical distribution of the main trophic groups catalyzing the process; **(c)** the engineered bioreactor system used to apply granules for wastewater treatment and biogas generation; **(d)** size distribution of biomass whereby granules were binned for this study into five size groups: extra-small (XS), small (S), medium (M), large (L), and extra-large (XL); and **(e)** the experimental set-up used to test granular growth where bioreactors were inoculated with either S, M, L, or the naturally distributed (mixed) biomass.

Granules were grouped (Figure 1) into five distinct size classifications: extra-small (XS; Ø, < 0.6 mm), small (S; Ø, 0.6–1.0 mm), medium (M; Ø, 1.0–1.4 mm), large (L; Ø, 1.4–1.8 mm), and extra-large (XL; Ø, > 1.8 mm). Granules were size-separated by passing the biomass through stainless steel sieves, separating specific size ranges. Triplicate samples from each size were stored at −20°C for subsequent DNA extractions. The remainder of the size-separated biomass was subsequently stored in phosphate-buffered saline (PBS) solution under an N_2_ headspace at 4°C prior to bioreactor inoculation.

### Bioreactor Design and Operation

Twelve, identical laboratory-scale (2 L) glass, EGSB bioreactors (Supplementary Figure S1) were constructed, and operated in four sets of triplicates: the first set (R_S1_–R_S3_) containing only S granules; the second set (R_M1_–R_M3_) containing only M-sized granules; the third set (R_L1_–R_L3_) containing only L granules; and the fourth set (R_N1_–R_N3_) started with the unfractionated, naturally distributed (N) biomass (Figure 1).

Apart from granule size in the starter biomass, the 12 bioreactors, each inoculated with 15 gVS L_bioreactor_^–1^, were operated identically at a 24 h HRT for 51 days. The biomass was allowed a 48-h acclimatization period at 37°C, regulated using built-in water jackets and recirculating water baths (Grant Optima, T100-ST12; Grant Instruments Ltd., Shepreth, United Kingdom), before feeding and recirculation were commenced, which were controlled using peristaltic pumps (Watson and Marlow 2058 and 300 series, respectively; Cork, Ireland). Influent was introduced at the base of each bioreactor at an OLR of 15.7 g COD L^–1^ day^–1^, and bioreactor liquor was recirculated through the system to achieve the superficial upflow velocity of 1.2 m/h according to the same set-up, and approach, as described previously (Collins et al., 2005; Madden et al., 2014).

The saccharide-rich, synthetic wastewater, based on recommendations and component ratios previously reported (Ahn and Forster, 2000), consisted of glucose (3.75 g/L), fructose (3.75 g/L), sucrose (3.56 g/L), yeast extract (1.45 g/L), and urea (2.15 g/L). The synthetic wastewater was further supplemented with trace elements (Shelton and Tiedje, 1984), and supplied to the 12 bioreactors from a single, thoroughly mixed reservoir to ensure homogeneity. Sodium bicarbonate (10 g/L) was added to the influent on day 6, and for the remainder of the experiment to act as a pH buffer, as the pH of the bioreactor liquor had dropped to 4 during the first week. This modification stabilized the influent pH at 7.8 for the remainder of the trial.

Upon take-down, on day 51, the biomass wet-weight was recorded, to determine total biomass loss or gain, and the entirety was re-fractionated into XS, S, M, L, and XL fractions to determine new size distribution. Samples from each size fraction were stored at -20°C for DNA extractions and sequencing.

### Sampling and Analytical Techniques to Monitor Bioreactor Performance

Biogas concentrations of methane, and effluent concentrations of total COD (tCOD), soluble COD (sCOD), volatile fatty acids (VFA), and pH, were monitored three times a week throughout the 51-day trial. Biogas methane concentrations were determined using a VARIAN CP-3800 gas chromatograph (Varian, Inc., Walnut Creek, CA, United States; details available in Supplementary Material). Methane yield efficiency was calculated using the theoretical yield based on COD and reported as an average over three operational phases. pH was measured using a benchtop meter (Hanna Instruments, Woonsocket, RI, United States). COD was measured using pre-prepared COD test kits (Reagacon, Shannon, Ireland) and following the recommendation of the manufacturer. Samples for tCOD assays were each prepared by adding a homogenous sample directly to the test kit, while for sCOD, the sample was first centrifuged for 10 min at 14,000 r/min and the supernatant was added to the test kit. COD tests were incubated for 2 h at 150°C and concentrations were determined using a spectrophotometer (Hach Dr/4000; Hach Company, Loveland, CO, United States) at 435 nm. VFA contents of supernatant from effluent samples were separated, and quantified, using gas chromatography (Varian 450-GC; Varian, Inc., Walnut Creek, CA, United States; details available in Supplementary Material).

### DNA Extraction

For each sample investigated, a mass of 0.1 g wet sludge was transferred to respective, sterile tubes in triplicate. Genomic DNA was extracted on ice following the DNA/RNA co-extraction method (Griffiths et al., 2000), which is based on bead beating in 5% (w/v) cetyl trimethylammonium bromide (CTAB) extraction buffer, followed by phenol-chloroform extraction. Quality of nucleic acids was assessed using a NanoDrop^TM^ spectrophotometer (Thermo Fisher Scientific, Waltham, MA, United States), and concentrations were determined using a Qubit fluorometer (Invitrogen, Carlsbad, CA, United States) and normalized to 5 ng DNA μL^–1^ for storage at -80°C.

### High-Throughput Gene Sequencing

Partial 16S rRNA gene sequences were amplified using the universal bacterial and archaeal primers, 515F and 806R, and under to the conditions previously applied by Caporaso et al. (2011), but using 2x KAPA HiFi HotStart ReadyMix (Roche; Clarehill, Clare, Ireland). After clean-up using an AMPure XP purification kit (Beckman Coulter, Clare, Ireland), according to the manufacturer’s instructions, amplicons were sequenced, with PhiX (PhiX Control Kit v3) as internal control, on an Illumina MiSeq platform (at FISABIO, Valencia, Spain).

### Bioinformatics and Statistical Analysis

Abundance tables were generated by constructing OTUs. An OTU table was generated for this study by matching the original barcoded reads against clean OTUs (a total of 2,793 OTUs for *n* = 49 samples) at 97% similarity (a proxy for species-level separation). Statistical analyses were performed in R (v. 3.4.4) using the combined data generated from the bioinformatics as well as meta data associated with the study. Alpha diversity analyses included the calculation of Shannon entropies and rarefied richness. Further multivariate integration (MINT)–sparse projection to latent structure (sPLS) algorithms identified study-wise discriminants. Additional details are available in Supplementary Material.

## Results

### Bioreactor Performance

**FIGURE 2:**
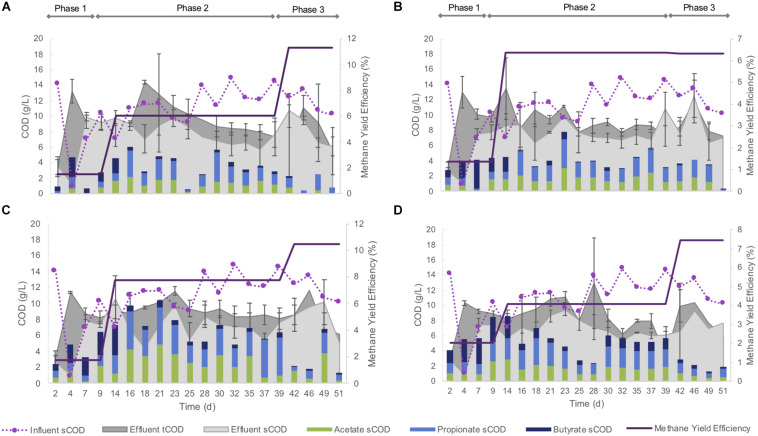
Methane yield efficiency; COD conversions (*n* = 3); and key VFA (acetate, propionate, and butyrate) contributions to effluent sCOD; in each of the four bioreactor sets: **(A)** R_S1_–R_S3_; **(B)** R_M1_–R_M3_; **(C)** R_L1_–R_L3_; **(D)** R_N1_–R_N3_.

Each of the four size-constrained sets of bioreactors responded similarly throughout the trial, regardless of being inoculated with differently sized granules. During the 4 days, influent pH decreased to 4.1 in each of the bioreactors. After supplementation of the influent with sodium bicarbonate, the pH stabilized (mean, pH 7.8) over the remainder of the experiment. Biogas methane concentrations were low during the initial acidification, but increased throughout the rest of the trial (Figure 2).

This pH shock also produced a strong effect on the COD removal. During the first 25 days of operation, more COD left the bioreactors than was supplied to them (Figure 2)—indicating biomass washout and a very unstable performance. This was largely reversed over the remainder of the trial, and COD removal, and methane yield efficiency continued to improve over the subsequent weeks, culminating in roughly 50% sCOD removal efficiencies by each of the bioreactors. Nonetheless, COD removal was lower again during the final approximately 2 weeks of the trial (Figure 2). Acetate, propionate, and butyrate contributed to 50–90% of effluent sCOD (Figure 2).

Biomass washout was observed from each bioreactor variously over the course of the 51-day experiment (Supplementary Figure S2), including from the “naturally distributed” condition (R_N1–3_). Bioreactor R_N2_ failed—and was stopped—on day 22, due to the loss of 52% of the biomass. The remaining 11 bioreactors experienced losses reaching up to 50%. Washout of biomass was noted particularly during the initial few days during the initial acidification, and again at the end of the trial, evidenced by increased COD in the effluent. A net gain in biomass was observed in only two bioreactors, R_L1_ and R_L3_.

### Shifts in Granule Size Distribution

**FIGURE 3:**
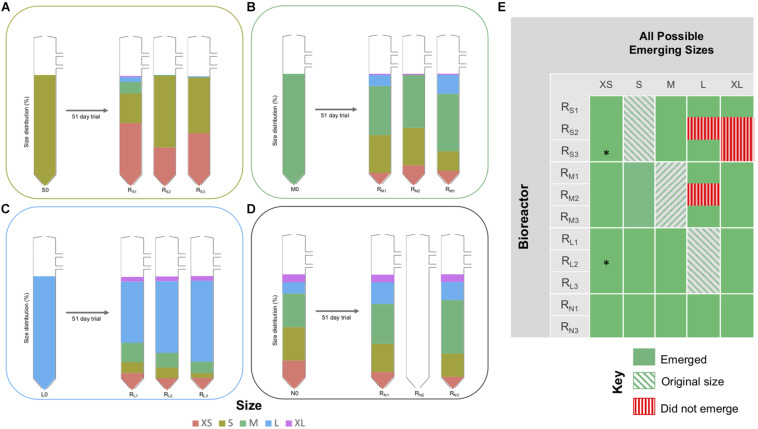
Changes in distribution of granule sizes in the R_S_, R_M_, R_L_, and R_N_ bioreactors during the trial (day 0 and each of the respective bioreactors at day 51), showing: **(A)** R_S1_–R_S3_; **(B)** R_M1_–R_M3_; **(C)** R_L1_–R_L3_; **(D)** R_N1_ and R_N3_ bioreactors. Colors indicate the granule size classification and their proportion of the total biomass present. **(E)** Map indicating frequency of observations of emerging sizes across the experiment. No sequencing data available for samples marked with (*).

Operation of laboratory-scale bioreactors, inoculated with size-constrained granules, allowed the emergence of “new” granule sizes, to be detected and studied. Size fractionation of biomass at the conclusion of the trial showed that the distribution of granule sizes had changed, and new granules—or “emerging sizes”—were apparent in all of the bioreactors (Figure 3). In all three of the R_L_ bioreactors, and in two of the R_M_ bioreactors, a full range of sizes (from the XS, S, M, L, XL classifications) had emerged (Figure 3). In the two surviving R_N_ bioreactors, granules each of the five size classifications were still present, although the proportion of granules in M or above had increased. In fact, with only the exception of L granules in R_S2_ and R_M2_, and XL granules in R_S2_ and R_S3_, all five sizes emerged from all bioreactors (Figure 3).

### Microbial Community Structure of New Sizes

**FIGURE 4:**
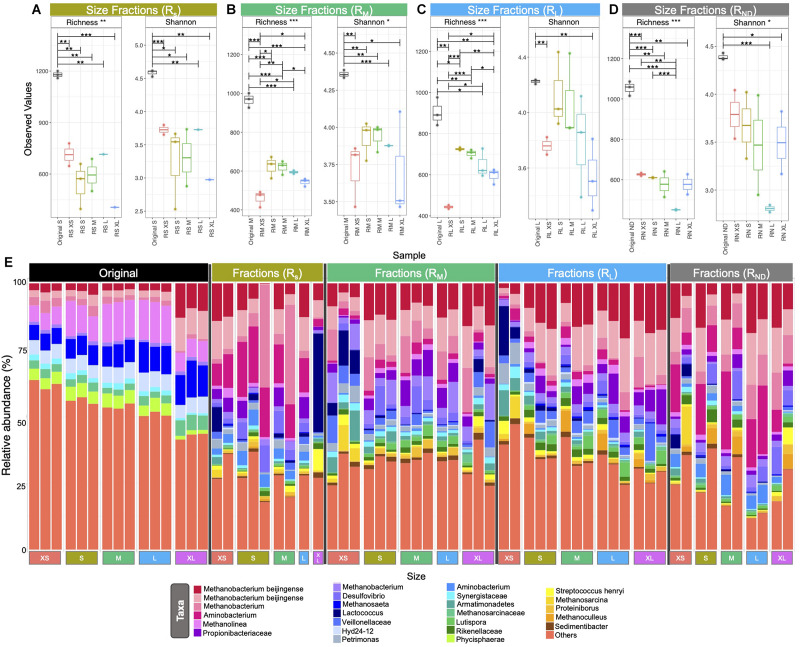
Box plots **(A–D)** of rarefied richness of the various size classifications from across the four bioreactor sets: **(A)** R_S1_–R_S3_; **(B)** R_M1_–R_M3_; **(C)** R_L1_–R_L3_; **(D)** R_N1_ and R_N3_; and bar chart **(E)** showing the top 25 relatively most abundant OTUs in original and new granules. Lines for figures **(A–D)** connect samples where differences were significant (ANOVA) indicated by *p < 0.05, **p < 0.01, ***p < 0.001.

Alpha diversity measurements, using Shannon entropy, indicated similar trends for granules from the R_M_ and R_L_ bioreactors (Figure 4). A reduction in alpha diversity was apparent from S through to XL granules (i.e., there was more diversity in the microbial communities found in S granules than in bigger ones). Nonetheless, the alpha diversity in XS granules was significantly lower than in S granules. In fact, the diversity found in XS granules was similar to the diversity in XL granules (Figure 4). Size fractions emerging from R_S_, however, were statistically similar, and did not follow the same diversity trend.

The initial (day 0) community structure comprised of a mix of hydrogenotrophic (*Methanobacterium*, *Methanolinea*) and acetoclastic (*Methanosaeta*) methanogens (archaea). At the same time, the bacteria found to be relatively most abundant were generally all heterotrophic fermenters. Over the course of the trial, the make-up of the most abundant taxa shifted considerably. Across all of the new (or growing) granules—i.e., the emerging sizes from the bioreactors—the community structure was dominated by four operational taxonomic unit (OTU) classifications of *Methanobacterium*, in many cases accounting for 25–50% of the relative abundance of all taxa (Figure 4). Interestingly, *Methanosaeta* completely disappeared from among the 25 most abundant OTUs. Other highly abundant taxa included *Aminobacterium*, *Propionibacteiraceae*, and *Desulfovibrio*.

Multivariate integration algorithms used for study-wise discriminant analyses identified a total of 38 “discriminant” OTUs from 11 distinct phyla using two identified “components” (Supplementary Figure S3). Mean relative abundances of these OTUs showed two general groupings: (i) those OTUs more abundant in either, or both, of the XS and XL sized granules, and (ii) those OTUs which were more abundant in the S, M, and L granule sizes.

## Discussion

### Emerging Sizes: Granules Grow

**FIGURE 5:**
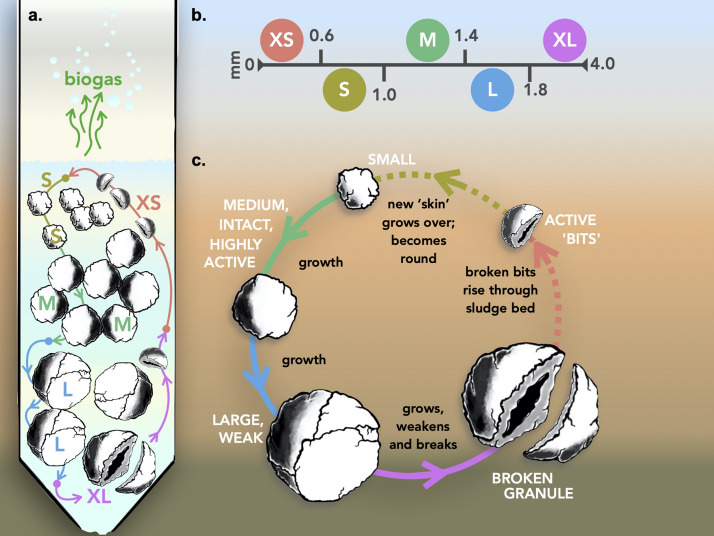
Granular growth and biofilm development model. **(a)** Operation of the model inside an anaerobic bioreactor; **(b)** size fraction parameters; and **(c)** the generalized growth model including the break-up of larger granules to form new, smaller granules.

This study demonstrates that methanogenic granules in anaerobic digesters do, indeed, “grow.” In each of the nine bioreactors started up with granules from a discrete size classification (Figure 1), the final distribution of granule sizes shifted to include new (or “grown”) granules that were either larger or smaller than the original granules (Figure 3), while also still containing granules of the original sizes. The emergence of larger granules almost certainly indicates the growth of granules due to cell replication and the accumulation of formerly planktonic cells from the surrounding environment. The observation of granules smaller than the original biomass might be explained in two ways: that (i) completely new granules formed from planktonic cells in the wastewater and the granulation process was continually initiated inside the digester, or (ii) parts of older, larger granules broke away and provided the foundation for new, small granules (Figure 5). The second explanation offers a potential mechanism of granule development. What is actually likely, we suggest, is that both phenomena proceed simultaneously.

An important component of the experiment was the set of bioreactors (R_N_) started up with a full complement of granule sizes, representing a “meta-community” of individual ecosystems (individual granules)—inspired in part by the recent description (Rillig et al., 2017) of soil aggregates as parallel incubators of evolution. In the R_N_ bioreactors, the relative size distribution shifted toward larger granules. This may be due to granule growth. Conversely, it may be that small granules were preferentially lost from R_N_ bioreactors, thus shifting the relative size distribution. However, smaller granules remained abundant in the other (R_S_, R_M_, or R_L_) bioreactors and many XS granules appeared to resist washout, ultimately suggesting that the shift in size distribution toward larger sizes was the result of growth.

Notably, the shift in size, and breaking of granules, while likely a natural process, was potentially further facilitated by the stressful conditions inside the bioreactors, similar to observations made regarding granule disintegration under high VFA conditions (McHugh et al., 2006). Moreover, we may also consider that the hydrodynamic conditions with respect to bioreactor scale (Connelly et al., 2017) and HRT (Arcand et al., 1994) may also have contributed to changes in the size distribution.

### Emerging Pathways: Dominance of Hydrogenotrophic Methanogenesis

Early in the trial the reactors were subject to a severe drop in pH, which although not ideal, grants insights into the microbial community of anaerobic granules under stress. The predominant members of the microbiome across all of the samples from the end of the trial included *Methanobacterium*, *Aminobacterium*, *Propionibacteriaceae*, and *Desulfovibrio* species—none of which were dominant initially. The shift in community was likely a consequence of the pH stress early in the trial. Previous studies (Hori et al., 2006) found that low pH and increasing VFA concentrations in anaerobic digesters resulted in more abundant *Methanosarcina* (acetoclastic and hydrogenotrophic methanogens) and *Methanothermobacter* (hydrogenotrophic methanogens) but fewer *Methanoculleus* (also hydrogenotrophic), concluding that VFA accumulation strongly influences archaeal community structure.

This was also supported by our study. *Methanosaeta (Methanothrix)*—an acetoclastic methanogen, which was abundant in the granules on day 0—was not detected in the new granules, while *Methanobacterium*—autotrophic, H_2_-using methanogens (Jarrell et al., 1982; Pennings et al., 1998; Shlimon et al., 2004; Ma et al., 2005; Lennon and Jones, 2011) also capable of formate reduction (Schauer and Ferry, 1980)—were dominant and likely feeding on increased dissolved hydrogen resulting from the accumulating VFA (Cord-Ruwisch et al., 1997). *Propionibacteriaceae*—a family of heterotrophic glucose fermenters, producing propionate and acetate as primary products (Akasaka et al., 2003)—were also abundant in new granules, likely as VFA-producing acetogens. It is, of course, interesting to observe that granules grew in this experiment without the apparent dominant involvement of the filamentous *Methanosaeta* (*Methanothrix*), which tends to contradict the conventional understanding of granulation microbiology (Weigant, 1987; Hulshoff Pol et al., 2004). Other filamentous bacteria, however, were present such as *Streptococcus*, which was recently linked to granulation in high-salinity wastewaters (Gagliano et al., 2020).

### Emerging Ecology: Supporting Syntrophic Relationships

The dominance of hydrogenotrophic methanogens (Supplementary Figure S3) by the end of the trial appeared to support the abundance of syntrophic bacteria, including *Aminobacterium*—heterotrophic fermenters of amino acids that grow well with methanogenic, H_2_-consuming partners, such as *Methanobacterium* (Baena et al., 1998; Hamdi et al., 2015)—and *Desulfovibrio*—sulfate-reducing bacteria (SRB) widespread in the environment (Goldstein et al., 2003), where they respire hydrogen or organic acids (Heidelberg et al., 2004) often in syntrophy with methanogens (Meyer et al., 2013). Interspecies metabolite exchange and hydrogen transfer (Stams and Plugge, 2009) between syntrophic partners is critical in AD because the oxidation of organic acids and alcohols by acetogens may be thermodynamically feasible only when hydrogenotrophic methanogens (in this case, likely the *Methanobacterium*) consume, and maintain sufficiently low concentrations of, H_2_. It is clear that the microbial community responded to the prevailing environmental stresses within the bioreactors. Indeed, had there not been an accumulation of VFA in the bioreactors and a striking dominance of the H2-oxidizing methanogens, a different community—perhaps characterized more strongly by the acetoclastic methanogens, may have developed.

### Emerging Discriminants: Size-Specific OTUs

In general, the communities of all differently sized granules were very similar with some, though few, significant differences in alpha diversity and rarefied richness. Nonetheless, 32 study-wise discriminants could be identified, using MINT-sPLS analysis, which were responsible for minor community shifts across the different sizes from each bioreactor set. Phylogenetically, these discriminants formed two distinct clades—the first made up primarily of the phyla Firmicutes, Synergistetes, and Chloroflexi, and the second clade comprising of Proteobacteria, Spirochaetae, Bacteroidetes, and Euryarchaeota. Many of the discriminant OTUs were generally upregulated in the S, M, or L granules, or were upregulated in either or both XS and XL granules. For example, *Lactococcus*, a glucose fermenter and primary member of the lactic acid bacteria group, and *Stenotrophomonas*, a likely nitrate reducer, were both upregulated in XS and XL granules, but rare in S, M, and L granules. Conversely, other taxa, such as the *Phycisphaerae*, *Leptospiraceae*, and *Bdellovibrio*, were upregulated in the S, M, and L granules but infrequent in XS or XL granules.

### Granular Growth and Biofilm Fate

Granulation and the subsequent growth of granules, resulting in various size distributions is not a new concept (Arcand et al., 1994; Batstone and Keller, 2001; Hulshoff Pol et al., 2004; Show et al., 2004), nor is it unique to anaerobic granules (Volcke et al., 2012; Zhou et al., 2016). Granular growth, which was clearly evidenced in our study, was also accompanied, and reported here for the first time, by the accumulation of XS granules in each of the initially size-constrained bioreactors, supporting the idea that granules break apart into smaller aggregates at some point during their “lifetime.” These smaller pieces did not appear to be selectively washed away, but were retained, making up a group of “new” smaller granules, which, in turn, will continue to grow. Such a concept is supported by previous evidence, that the larger the granule becomes, the more structurally unstable it is (Díaz et al., 2006), and that it eventually breaks apart. Our idea is that these broken bits, still containing an active microbial community eventually round off (due to shear forces within the digester) and become the basis for new, small granules, so that the process is cyclical (Figure 5).

To accept such a concept regarding granular growth, we would need to see that bioreactors initially containing only small granules, would eventually contain medium, then large and, finally, XL granules. An equivalent scenario would be observed for each bioreactor set. Equally, clear trends in microbial community structure might be observed across the different sizes. For example, an XL granule would have a similar community structure to an XS granule, but may be significantly different to an S or M granule.

Although the experimental design provided an interesting means to uncover the trajectory and fate of granules, each set of bioreactors was started with a different, size-limited, microbial consortium. Thus, granules grew from size-constrained consortia rather than a replete reservoir of granule sizes. Nonetheless, this study does provide evidence for “growing” granules and for the emergence and retention of very small granules, which are either the result of bigger aggregates breaking apart, or continued growth of *de novo* granules—but likely both. Granule growth was apparent in all nine of the R_S_, R_M_, and R_L_ bioreactors. Indeed, most contained granules—albeit, sometimes very few—from each of the five size classifications used. Moreover, this study would suggest, based on emergence of XS granules in the R_S_ bioreactors (Figure 3), that even small granules can break apart.

## Conclusion

In summary, granules were demonstrated to be dynamic aggregates inside anaerobic digesters, appearing to follow a progressive growth pattern from small, to medium to large. XS granules emerged in all bioreactors, regardless of the starting size distribution. These either formed *de novo*, from the aggregation of free cells, or as a result of larger granules breaking apart. Further experiments should be done, under more stable bioreactor conditions, and with more intensive sampling regimes, to provide more evidence. The rate of biomass accumulation, as well as requirements to replace biomass, impinge on bioreactor performance and are important considerations in biomass management in anaerobic digesters. The results of experiments based on innovative approaches to track the fate of growing granules will provide valuable information to environmental engineers running bioreactors and to microbial ecologists studying community assembly phenomena, alike.

## Author’s Note

This manuscript has been released as a preprint at BioRxiv (Trego et al., 2019).

## Data Availability Statement

The sequencing data from this study are available on the European Nucleotide Archive under the study accession number PRJEB28212 (http://www.ebi.ac.uk/ena/data/view/PRJEB28212).

## Author Contributions

AT, SC, UI, and GC designed the study. AT performed all of the physico-chemical characterization with assistance from CM, SM, EG, CS, SD, and CN. AT prepared the sequencing libraries. UI wrote the scripts for data analysis, which was conducted by AT. Results were interpreted by AT, CQ, UI, and GC. AT drafted the manuscript. UI and GC revised the document. All authors approved the manuscript and agreed for accountability of the work therein.

## Conflict of Interest

The authors declare that the research was conducted in the absence of any commercial or financial relationships that could be construed as a potential conflict of interest.
