# Supplementary material for: Growth and Break-Up of Methanogenic Granules Suggests Mechanisms for Biofilm and Community Development
Source: Front Microbiol. 2020 Jun 3;11:1126. doi: 10.3389/fmicb.2020.01126 (PMC7285868; doi:10.3389/fmicb.2020.01126)
Supplement: Supplementary file 1 [file Data_Sheet_1.docx]

Research Article

**GROWTH AND BREAK-UP OF METHANOGENIC GRANULES SUGGESTS MECHANISMS FOR BIOFILM AND COMMUNITY DEVELOPMENT**

Trego et al.

**SUPPLEMENTAL MATERIALS AND METHODS**

**Analytical techniques to monitor bioreactor performance.** VFA and methane concentrations were measured using gas chromatographs (GC). For VFA concentrations, the GC was equipped with a Combi PAL auto-sampler system (Varian, Inc., Walnut Creek, CA) and a 25-m long, CP-WAX 58 (FFAP) Capillary column (SGE Analytical Sciences) with an internal diameter of 0.32 mm and 0.2 μm film thickness. Liquid samples in 1 ml aliquots were injected into the front injector (port type 1177) at 250ºC. Helium gas carried the sample at a flow rate of 1 ml min^-1^ under a constant flow and pressure of 0.1 psi. Oven temperature was operated as follows: 60ºC for the first 0.1 min, 100ºC for 1.17 min, and 200ºC for 12.97 min. VFA were detected using a flame ion detector (FID) at 300ºC and identified and quantified by assigning chromatographic retention times against a calibration curve of known VFA. 2-Ethylbutyric acid was used as the internal standard.

The method for methane detection used a stainless-steel column filled with HaveSep® C polymer. The injection port and hydrogen flame ionisation detection temperatures were at 100ºC and 105ºC, respectively. Nitrogen was used as the carrier gas at a constant flow rate of 30 ml min^-1^. Data were recorded through the Varian Star Chromatography Workstation (version 6.2) integration software.

**Bioinformatics and statistical analysis.** Sickle v1.200 (Joshi and Fass, 2011) was used to trim and filter paired-end reads. This software used a sliding window technique and trimmed regions where the average base quality dropped below 20. Next, a length threshold of 10 bp was used to discard reads that fell below this length. BayesHammer (Nikolenko et al., 2013) was applied from the Spades v2.5.0 assembler, which error-corrected the paired-end reads. Following this, pandaseq v(2.4) was used to assemble the forward and reverse reads into a single sequence spanning the entire V3-V4 region with a minimum overlap of 20 bp. This provided consensus sequences for each sample. Recent work (Schirmer et al., 2015; D’Amore et al., 2016) has shown that this pipeline significantly reduces substitution rates (which is the primary type of error encountered in datasets generated by the Illumina MiSeq platform).

Next, VSEARCH (v2.3.4) was used for OTU construction (the steps are documented at http://github.com/torognes/vsearch/wiki/VSEARCH-pipeline). First, all reads from each sample were pooled together while barcodes were added to keep track of from which sample the read originated. The reads were then de-replicated, sorted in order of decreasing abundance, and singletons were discarded. Next, the reads were clustered based on 97% similarity, followed by removing clusters with chimeric models built from more abundant reads (--uchime_denovo option in vsearch). To remove any chimeras that may have been missed, particularly in the case that they had parents that were absent from the reads or were present in very low abundance, a reference-based chimera filtering step (--uchime_ref option in vsearch) using a gold database (https://www.mothur.org/w/images/f/f1/Silva.gold.bacteria.zip) was applied.

The assign_taxonomy.py script from the Qiime workflow (Caporaso et al., 2010) was used to taxonomically classify the representative OTUs against the SILVA SSU Ref NR database release v123 database. Phylogenetic distances between OTUs were resolved using kalign v2.0.4 (Lassmann and Sonnhammer, 2005) as a multisequence alignment (options –gpo 11 –gpe 0.85). Following this, FastTree v2.1.7 (Price et al., 2010) generated the phylogenetic tree in NEWICK format and biome files for the OTUs were generated by combining the abundance table with taxonomy information using make_otu_table.py from the Qiime workflow.

The vegan package (Oksanen et al., 2015) was used for alpha and beta diversity analyses. For alpha diversity measures we used: **(i)** *rarefied richness* – the estimated number of species/features in a rarefied sample (to minimum library size); and **(ii)** *Shannon entropy* – a commonly used index to measure balance within a community. Additionally, a study-wise discriminant-out-based comparison of reactor set-ups (R_S_, R_M_, R_L_, and R_N_) between emerging fractions (XS, S, M, L, and XL) was applied. The Multivariate Integration (MINT) algorithm (Rohart et al., 2017) was used, which is an extension of the multi-group Projection to Latent Structure (mgPLS), and attempts to find a common projection space across all studies, defined on a small subset of discriminative variables which consistently discriminate the outcome classes. MINT combined $M=4$ datasets denoted $X^{\left( 1 \right)}\left( N_{1}\times P \right)$, $X^{\left( 2 \right)}\left( N_{2}\times P \right)$,…, $X^{\left( 4 \right)}\left( N_{4}\times P \right)$ for *R_L_,* *R_M_*, *R_N_*, and *R_S_* respectively, where all the datasets share the *P* OTUs whilst the number of samples differ, i.e., $N_{1}$, $N_{2}$ ,…,$N_{4}$. All studies associated dummy indicator outcome $Y^{\left( 1 \right)}$, $Y^{\left( 2 \right)}$,…, $Y^{\left( 4 \right)}$ in which all the emerging fractions (XS, S, M, L, and XL) are represented. MINT then solved the problem: ${\text{max} \atop a_{h}, b_{h}}\sum_{m=1}^{M} N_{m}\text{cov}(X_{h}^{(m)}a_{h},Y_{h}^{(m)}b_{h})$, such that $\left\| a_{h} \right\|_{2}=1$ and $\left\| a_{h} \right\|_{1}\leq\lambda$, where the covariance of scores between the datasets are maximised by identifying the global loading vectors $a_{h}$ and $b_{h}$ common to all studies. Here, $h$ represents the number of components (akin to PCA analysis). Since there is a sparsity control parameter $\lambda$ in the above equation, adjusting it enforces shrinkage and any non-zero associated weights in the global loading vector $a$ are able to identify discriminant OTUs in a global sense between the outcome classes. The MINT algorithm was used on the OTUs according to recommendations given at <http://www.mixomics.org> where after pre-filtering 1% of the OTUs in lowest abundance, TSS+CLR (Total Sum Scaling followed by Centralised Log Ratio) normalisation was performed prior to the application of the MINT algorithm.

**References**

Caporaso, J. G., Kuczynski, J., Stombaugh, J., Bittinger, K., Bushman, F. D., Costello, E. K., et al. (2010). QIIME allows analysis of high-throughput community sequencing data. *Nat. Methods* 7, 335. Available at: http://dx.doi.org/10.1038/nmeth.f.303.

D’Amore, R., Ijaz, U. Z., Schirmer, M., Kenny, J. G., Gregory, R., Darby, A. C., et al. (2016). A comprehensive benchmarking study of protocols and sequencing platforms for 16S rRNA community profiling. *BMC Genomics* 17, 55. doi:10.1186/s12864-015-2194-9.

Joshi, N., and Fass, J. (2011). Sickle: A sliding-window, adaptive, quality-based trimming tool for FastQ files (Version 1.33).

Lassmann, T., and Sonnhammer, E. L. L. (2005). Kalign -- an accurate and fast multiple sequence alignment algorithm. *BMC Bioinformatics* 6, 298. doi:10.1186/1471-2105-6-298.

Nikolenko, S. I., Korobeynikov, A. I., and Alekseyev, M. A. (2013). BayesHammer: Bayesian clustering for error correction in single-cell sequencing. *BMC Genomics* 14, S7. doi:10.1186/1471-2164-14-S1-S7.

Oksanen, J., Blanchet, F., Kindt, R., Legendre, P., Minchin, P. R., O’hara, R., et al. (2015). Vegan: community ecology package. R Package version 2.2-1.

Price, M. N., Dehal, P. S., and Arkin, A. P. (2010). FastTree 2 – Approximately Maximum-Likelihood Trees for Large Alignments. *PLoS One* 5, e9490. Available at: https://doi.org/10.1371/journal.pone.0009490.

Rohart, F., Gautier, B., Singh, A., and Lê Cao, K.-A. (2017). mixOmics: An R package for ‘omics feature selection and multiple data integration. *PLOS Comput. Biol.* 13, e1005752. Available at: https://doi.org/10.1371/journal.pcbi.1005752.

Schirmer, M., Ijaz, U. Z., D’Amore, R., Hall, N., Sloan, W. T., and Quince, C. (2015). Insight into biases and sequencing errors for amplicon sequencing with the Illumina MiSeq platform. *Nucleic Acids Res.* 43. doi:10.1093/nar/gku1341.
