# Supplementary material for: Growth and Break-Up of Methanogenic Granules Suggests Mechanisms for Biofilm and Community Development
Source: Front Microbiol. 2020 Jun 3;11:1126. doi: 10.3389/fmicb.2020.01126 (PMC7285868; doi:10.3389/fmicb.2020.01126)
Supplement: Supplementary file 2 [file Data_Sheet_2.docx]

Research Article

**GROWTH AND BREAK-UP OF METHANOGENIC GRANULES SUGGESTS MECHANISMS FOR BIOFILM AND COMMUNITY DEVELOPMENT**

Trego et al.

**SUPPLEMENTAL FIGURES**


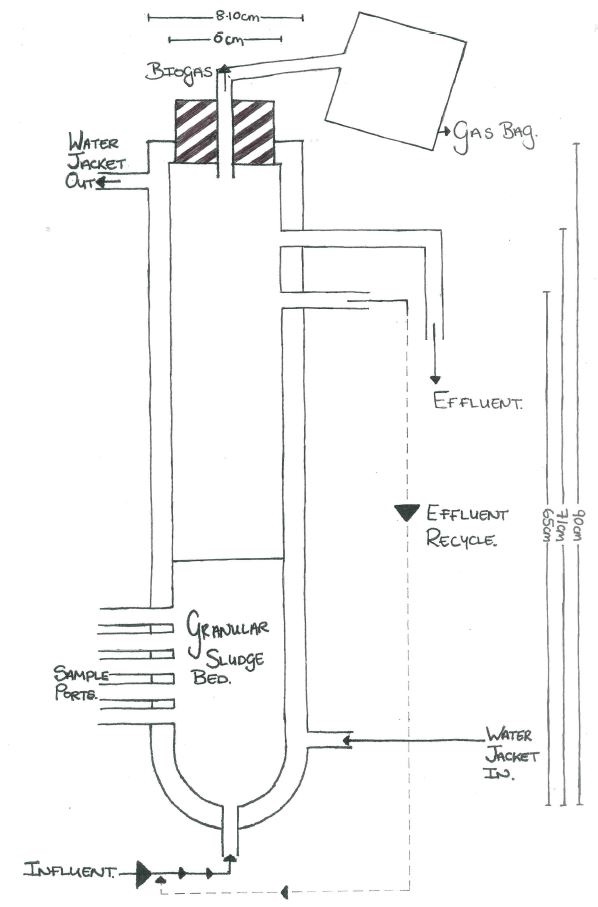


**Figure S1.** Schematic of the EGSB bioreactors employed during the 51-day trial.

**Figure S2.** Bar plot of the biomass yield, calculated on d-51, for each bioreactor, where (*) indicates the bioreactor that was shut down after day 22 due to high biomass losses

**Figure S3.** MINT study-wise discriminant analysis where **(a)** shows the first two components of samples (MINT PLS-DA) using all the OTUs with ellipse representing 95% confidence interval and percentage variations explained by these components in axes labels; **(b)** shows the optimum number of discriminating OTUs found for these 2 components identified as diamonds; and **(c)** is similar to **(a)** but the samples are drawn only using the 38 discriminant OTUs (MINT sPLS-DA); (**d – g**) show the MINT sPLS loading vectors $a_{1}$ and $a_{2}$ with non-zero weights for component 1 and component 2 where **(d)** shows contributions by emerging granules from R_S_; **(e)** from R_M_; **(f)** from R_L_ and **(g)** from R_N_ studies. Loading vectors are coloured by emerging size with maximal abundance (note: while interpreting this figure, focus should be on the colour of the bars and not the positive/negative projections); **(h)** the heatmap with mean relative abundance values (drawn using EvolView <http://www.evolgenius.info/evolview/>); and **(i)** the taxonomic classification of discriminant OTUs coloured by unique phyla to which they belong

**Figure S4.** Top 25 most abundant taxa from the emerging sizes, ordered by size and based up on variances in the 16S rRNA gene
